# The Effects of Anthocyanins Added to Semen Diluent on Semen Quality, Semen Antioxidant Capacity, and Sperm Apoptosis in Zi Geese

**DOI:** 10.3390/ani15223281

**Published:** 2025-11-13

**Authors:** Size Wang, Chuicheng Zeng, Puxuan Zhao, Yue Pan, Zhengyu Zang, Yuanliang Zhang, Shan Yue, Shengjun Liu, Peng Zheng, He Huang, Xiuhua Zhao

**Affiliations:** 1 Animal Husbandry Institute, Heilongjiang Academy of Agricultural Sciences, Harbin 150086, China; m15546580001@163.com (S.W.); zhangyuanliang1218@126.com (Y.Z.); ys924634716@126.com (S.Y.); 2 College of Animal Science and Technology, Northeast Agricultural University, Harbin 150030, China; 18236195872@163.com (C.Z.); 19832390209@163.com (P.Z.); 13019655610@163.com (Y.P.); zhengyuzang@126.com (Z.Z.); zhengpeng@neau.edu.cn (P.Z.); 3 College of Animal Science and Veterinary Medicine, Heilongjiang Bayi Agricultural University, Daqing 163319, China; lsj4396@163.com

**Keywords:** Zi goose, anthocyanins, semen quality, oxidative stress, apoptosis

## Abstract

The Zi goose is an excellent local goose breed in China, characterized by strong tolerance to roughage, high cold resistance, and high egg production. Anthocyanins are natural water-soluble pigments with numerous biological functions such as growth promotion, antioxidant activity, and immune regulation. However, there are few reports on whether anthocyanins have an improving effect on Zi goose semen. This study aimed to explore the effects of anthocyanins on semen quality, semen antioxidant capacity, and sperm apoptosis of Zi geese, so as to provide references for the large-scale breeding and industrial utilization of Zi geese. Sixty 12-month-old Zi geese were selected for the experiment, and their semen was collected by the massage method. Semen diluent containing different concentrations of anthocyanins was added to the mixed semen, which was then stored at 37 °C for detections. The results showed that the sperm survival rate of Zi geese was the highest when 30 mg/L of anthocyanins was added to the diluent. Compared with the control group, the anthocyanin group showed significantly higher sperm survival rate, sperm motility, plasma membrane integrity rate, acrosome integrity rate, mitochondrial activity, and DNA integrity (*p* < 0.05), while the sperm mortality rate was significantly decreased (*p* < 0.05). There were no significant differences in semen pH value, sperm density, and sperm abnormality rate (*p* > 0.05). The contents of superoxide dismutase and glutathione peroxidase were significantly increased (*p* < 0.05), while the levels of reactive oxygen species and malondialdehyde were significantly decreased (*p* < 0.05) in the anthocyanin group. Anthocyanins significantly increased the mRNA and protein expression of *Bcl-2* (*p* < 0.05), and significantly decreased the mRNA and protein expression of *Bax*, *Caspase-3*, and *P53* (*p* < 0.05). In conclusion, adding 30 mg/L anthocyanins to semen diluent can improve semen quality in Zi geese.

## Simple Summary

The protective effect and mechanism of anthocyanins on goose semen are not clear. This study aimed to explore the effects of anthocyanins on semen quality, semen anti-oxidant capacity, and sperm apoptosis of Zi geese. The addition of 30 mg/L of anthocyanins to the semen diluent improved semen quality and antioxidant capacity while reducing sperm apoptosis in Zi geese. These findings provide references for the large-scale breeding and industrial utilization of Zi geese.

## 1. Introduction

China has the richest goose breed resources in the world, as well as being the largest goose breeding and consumption country. The goose industry occupies an important position in husbandry. Zi goose, an excellent local small-egg-type goose breed in China, mainly lives in Heilongjiang Province. It has become one of the core breeds for egg goose breeding in the cold regions of northern China due to its prominent advantages such as early sexual maturity, excellent egg-laying performance, strong adaptability, and good tolerance to roughage [1]. Zi goose semen is mainly used for artificial insemination in production, which can not only significantly improve the utilization rate of male geese and reduce the number of male geese and costs, but also effectively avoid disease transmission during natural mating and ensure the health of geese [2]. With the popularization of large-scale breeding of Zi geese and the in-depth development of breed improvement, the low reproductive efficiency of Zi geese has become a major bottleneck restricting the intensive development of Zi geese. As a key indicator to measure the fertility of male geese, semen quality directly determines the success rate of artificial insemination and the fertilization rate of eggs. Therefore, research on the regulation of Zi goose semen quality has become a key direction in the current field of Zi goose reproduction research.

Anthocyanins are a class of water-soluble natural pigments widely present in plants, belonging to flavonoids, and are commonly found in plant tissues such as blueberries, blackberries, purple cabbage, and grape seeds [3]. Modern pharmacological studies have confirmed that anthocyanins have extremely strong antioxidant activity, with a free radical scavenging capacity 20 times that of vitamin C and 50 times that of vitamin E. They can inhibit lipid peroxidation reactions, scavenge reactive oxygen species (ROS) and reactive nitrogen species, and protect the structural and functional integrity of cell biomembranes [4]. Anthocyanins have a wide range of sources, low toxicity, and no residue risk, showing broad application prospects in the fields of food, medicine, and husbandry [5,6].

At present, certain research progress has been made on the regulation of semen quality in livestock by anthocyanins [7,8]; however, reports on Zi geese are still limited. A study by AlGhannam and El-Rahman found that an appropriate concentration of anthocyanins (10–20 mg/kg b.w) added to the diet could significantly improve sperm motility and sperm density in rats with infertility induced by AlCl3 [9]. A large number of studies have confirmed that anthocyanins can alleviate oxidative stress damage and maintain the oxidation–antioxidation balance by activating the antioxidant defense system [10,11]. Pawłowicz et al. found that anthocyanins can be useful in controlling oxidative stress in males with oligospermia [12]. Anthocyanins can reduce sperm apoptosis by regulating the expression of apoptosis-related genes [13]. Although the above studies provide a theoretical basis for the application of anthocyanins in the regulation of Zi goose semen, as a unique cold-region egg-type breed, the physiological characteristics and metabolic rules of Zi goose semen are significantly different from those of other livestock. Since the semen of Zi geese is susceptible to oxidative stress damage during preservation, leading to decreased quality, while anthocyanins possess strong antioxidant activity and can scavenge free radicals and protect the structure and function of sperm, this study was conducted to investigate the effect of adding anthocyanins to Zi geese semen diluent on semen quality.

Based on the above research background, Zi geese semen was taken as the research object. The purpose of this study was to screen out the optimal addition concentration of anthocyanins in Zi goose semen, clarify the action pathway of anthocyanins in the regulation of Zi goose semen quality, and provide a scientific basis for the development of safe and efficient natural protectants for Zi goose semen, thereby improving the artificial insemination efficiency and breeding value of Zi geese and promoting the sustainable development of the egg goose industry in the cold regions of northern China.

## 2. Materials and Methods

### 2.1. Experimental Design

This study collected semen from Zi geese, and qualified semen was selected for subsequent experiments after evaluating the quality of the semen. The experiment was divided into a control group and an anthocyanin group. The control group used a diluent without anthocyanins to dilute semen, while the anthocyanin group used diluents with 10, 30, 50, 70, and 90 mg/L of anthocyanins added to dilute semen, respectively. The optimal concentration of anthocyanins added to semen diluent was screened by comparing sperm survival rates. Subsequently, the semen in the anthocyanin group was diluted with a diluent containing the optimal concentration of anthocyanin. We compared the sperm survival rate, sperm motility, motility parameters, acrosome integrity rate, plasma membrane integrity rate, mitochondrial activity, ROS content, glutathione peroxidase (GPx) activity, malondialdehyde (MDA) content, superoxide dismutase (SOD) activity, sperm apoptosis level, and sperm DNA fragmentation index between the control group and the anthocyanin group. Each experiment was repeated at least three times. The effects of adding the optimal concentration of anthocyanins to semen diluent on the quality, antioxidant capacity, and sperm apoptosis of Zi geese were systematically explored.

### 2.2. Anthocyanins and Anthocyanins Solution Preparation

Anthocyanins extracted from blueberry were obtained from Harbin Chaofeng Biological development Co., Ltd. (Harbin, China; purity ≥ 25%). The required amount of anthocyanin powder was weighed and slowly added to a 1% citric acid solution at a ratio of 1:20. The solution was vortexed for 2 min until no visible particles were observed by the naked eye. The above anthocyanin solution was slowly added into the preheated semen diluent at 37 °C, gently stirring while adding. After the dropwise addition was complete, the pH value of the diluent was adjusted to 6.8–7.4. The anthocyanin-containing diluent was filtered through a 0.22 μm filter membrane and immediately used for subsequent operations. All steps were performed in a dark room under red light (wavelength 620–750 nm) with a black light-shielding mat laid on the operating table.

### 2.3. Experimental Animals

Sixty male Zi geese at 12 months of age were selected. The experimental period was from 1 April to 30 June 2025. The indoor temperature was maintained at 22 ± 3 °C and humidity at 55–65%, with a light cycle of 16 h. The geese were raised in a single pen and were allowed access to food and water ad libitum during the experiment. A one-week pre-experimental trial was conducted before the formal experiment began.

### 2.4. Semen Collection

Semen was collected using the massage method. Before semen collection, a two-week training period was conducted to exclude individuals with poor sexual response or inability to collect semen. Semen collection was performed between 8:00 AM and 12:00 PM in the pen. During collection, the male goose was held on a table with the tail facing the collector, and was massaged several times on its abdomen and back. Once the penis of male goose erected and extended from the cloaca, the ejaculated semen was collected using a semen cup. Semen from each goose was stored separately. Semen was collected every three days.

### 2.5. Semen Processing

After semen collection, the samples were placed in a 37 °C insulated cup and returned to the laboratory within 1 h. Semen with normal color, odor, and volume was selected and examined under a 400× microscope (Olympus BX53, Shinjuku, Tokyo, Japan). Only semen with sperm motility above 0.75 was chosen. The qualified semen was mixed uniformly, and 1 mL of semen was aliquoted into each 1.5 mL centrifuge tube. The semen was then diluted 100-fold using pre-warmed semen diluent at 37 °C for subsequent experiments. The composition of semen diluent is detailed in Table 1.

### 2.6. Evaluation of Sperm Quality

#### 2.6.1. Single Semen Volume

The semen volume was measured using a collection cup (GF-600, ZhengZhou Greide Agriculture and Animal Husbandry Technology Co., Ltd, ZhengZhou, China) with scale (accurate to 0.1 mL) after semen collection.

#### 2.6.2. Semen pH Value

The pH value of semen was measured using a pH meter (Orion Star A211, Thermo Fisher Scientific, Waltham, MA, USA) [14].

#### 2.6.3. Sperm Survival Rate

Firstly, 1.4 μL of the diluted semen was pipetted in a sperm counting chamber. Then, the chamber was placed on a 37 °C heated stage. Finally, sperm survival rate was determined using a computer-assisted sperm analysis system (CASA; Hamilton Thorne, Beverly, MA, USA) under a 100× phase-contrast microscope (ML-800, Songjing Tianlun Biotechnology Co., Ltd., Nanning, China). A total of no fewer than 500 sperms needed to be counted across all observed fields.

#### 2.6.4. Sperm Parameters

The sperm concentration, motility, abnormal rate, motility parameters (curvilinear velocity (VCL), straight-line velocity (VSL), and average path velocity (VAP)), acrosome integrity rate, and membrane integrity rate were analyzed using CASA [15].

### 2.7. Mitochondrial Activity Assay

Mitochondrial activity was assessed using the JC-1 and PI dual fluorescent staining method. An 80 μL semen sample was added to a pre-warmed centrifuge tube, followed by 4 μL of JC-1 and 2 μL of PI staining solution. The mixture was incubated in the dark for 30 min. Then, 10% of the total volume of Hancock’s solution was added, and the mixture was homogenized by pipetting. Subsequently, 10 μL of the mixed solution was placed on a glass slide, and the samples were observed under a 400× fluorescence microscope after cytocentrifugation. Five random fields were examined for each experimental group, ensuring at least 200 sperms per field.

### 2.8. Semen Antioxidant Capacity Detection

The sperm sample was centrifuged at 500× *g* for 5 min, then washed three times with pre-cooled sterile PBS solution. Subsequently, the semen was diluted to 1  ×  10^7^/mL and placed into 1.5 mL centrifuge tube [16]. Finally, the antioxidant indicators of semen were measured using the Beyotime ROS assay kit (Shanghai, China), GPx assay kit (NanJing JianCheng Bioengineering Institute, Nanjing, China), MDA assay kit (NanJing JianCheng Bioengineering Institute, Nanjing, China), and SOD assay kit (NanJing JianCheng Bioengineering Institute, Nanjing, China) according to Jing’s method [15].

### 2.9. Sperm Apoptosis Detection

Firstly, 100 μL of sperm suspension was added into each flow tube, then 5 μL of FITC-Annexin V and 10 μL of PI were added. The mixed solution was incubated in the dark for 15 min. Next, 400 μL of buffer was added, and the sample underwent flow cytometry analysis within 1 h to determine the apoptosis rate.

### 2.10. Relative mRNA Expression Detection

The mRNA expression was determined by real-time PCR as in a previous study [16]. The mRNA was extracted from sperm using TRIzol reagent following the method provided by the manufacturer (Invitrogen, Shanghai, China). The concentration of RNA was quantified using a NanoDrop^®^ lite (Thermo Fisher, Waltham, MA, USA). Complementary DNA (cDNA) was synthesized using a PrimeScript™RT reagent kit (TaKaRa, Katsushika, Japan) in a volume of 60 μL (containing 5 μg of the total RNA) following the manufacturer’s instructions. Synthesized cDNAwas diluted five-fold with sterile water and was stored at −20 °C until the next step. Quantitative real-time PCR was performed using LightCycler^®^ 96 (Roche, Life Science, Basel, Switzerland) with the SYBR^®^ PrimeScript™ RT-PCR Kit (Roche, Basel, Switzerland) according to the manufacturer’s instructions. Reactions were performed in a 20 μL reaction mixture containing 10 μL of the 2× SYBR Green I PCR Master Mix, 2 μL of cDNA, 0.4 μL of each primer (10 μM), and 7.2 μL of PCR-grade water. PCR procedure consisted of 95 °C for 10 min, followed by 40 cycles of denaturing–annealing/elongating (95 °C for 15 s and 60 °C for 1 min) and melting curve analysis (95 °C for 15 s and 60 °C for 20 s). The melting curve analysis showed only one peak for each PCR product. There were three duplications for each sample. The stability of GAPDH genes was evaluated by measuring the fluctuation range of the Ct values. The results were calculated using the 2^(−ΔΔCt)^ method. Primer information is detailed in Table 2.

### 2.11. Western Blot Detection

Semen was centrifuged and total protein was extracted using RIPA lysis buffer with PMSF protease inhibitors. The protein concentration was measured using the BCA method and standardized. SDS-PAGE gel was prepared, and the protein was separated by electrophoresis after sample loading and then transferred to PVDF membrane by wet printing. We sealed 5% skimmed milk for 1.5 h. *Bax*, *Bcl-2*, *Caspase-3*, and *P53* primary antibodies were added, incubated overnight at 4 °C. After TBST membrane washing, HRP-labeled secondary antibodies were added and incubated at room temperature for 1 h; ECL chemiluminescence was developed, and the band gray value was quantitatively analyzed by Image J software (National Institutes of Health, Bethesda, MD, USA). The references for antibodies and final concentrations during incubations used in this study are shown in Table S1.

### 2.12. DNA Fragment Index Detection

The semen was optimized and stained using a sperm nuclear integrity staining kit (NanJing Xindi Bioengineering Institute, Nanjing, China). A total of 5 μL of semen sample was mixed with 95 μL of LA solution, and 200 μL of acid treatment solution was added for 30 s. Then, 600 μL of staining solution was added and mixed well. After staining was completed, flow cytometry was used to detect 5000 sperm per sample. FL1 and FL3 data were collected, and DNA fragment index detection was performed [15].

### 2.13. Data Analysis

All experimental data from the present study are presented as mean ± standard deviation (SD). The data was recorded using Excel software and statistically analyzed using SPSS 26.0 software (version 26.0, Chicago, IL, USA). Comparison of two groups was performed using a two-way analysis of variance (ANOVA) with the Bonferroni–Dunn multiple comparison test. When the *p* value was less than 0.05, the groups were considered to have a significant difference.

## 3. Experimental Results

### 3.1. Semen Collection Situation

According to Table S2, semen was collected normally from 55 geese among the 60 experimental geese in this study, and the proportion of geese from which semen was collected was 91.67% of the total.

### 3.2. Effect of Semen Storage Time on Sperm Survival Rate of Zi Geese

**Figure 1:**
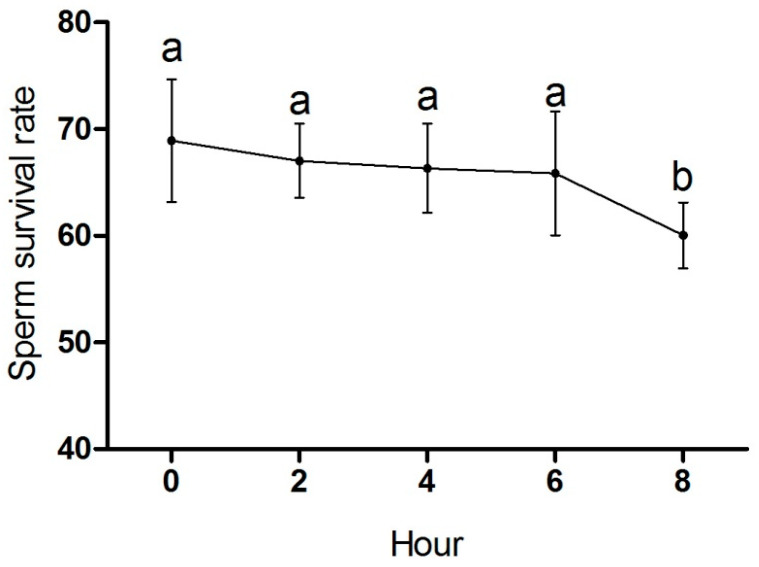
Sperm survival rate of Zi geese for 8 h preservation. Data are expressed as the mean ± SD (*n* = 10). The dots with different lowercase letters represent statistically significant differences between the groups (*p* < 0.05); the dots with a common lowercase letter are not significantly different (*p* > 0.05).

In order to investigate the effect of storage time on sperm survival rate, the semen was diluted 100 times and stored in a constant-temperature incubator at 37 °C. As shown in Figure 1, the sperm survival rate of the geese gradually decreased with the increase in storage time. The sperm survival rate significantly decreased after 8 h (*p* < 0.05). Therefore, diluted semen was stored for 6 h for subsequent experiments.

### 3.3. Effects of Different Anthocyanin Concentrations on Sperm Survival Rate of Zi Geese

**Figure 2:**
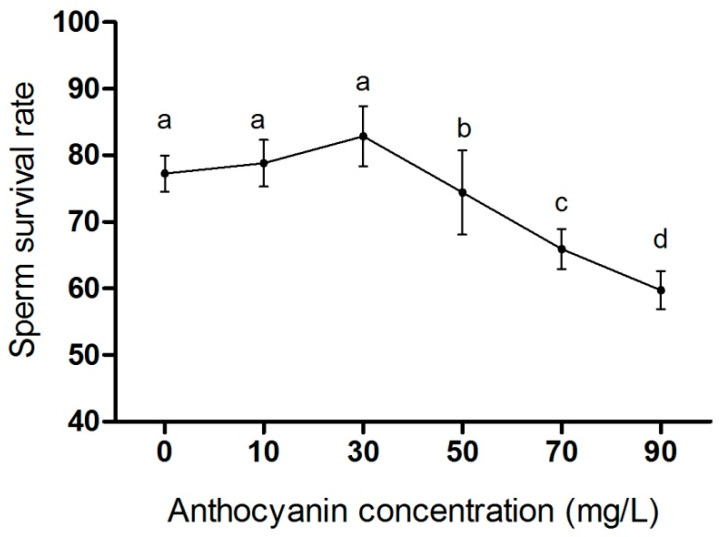
The effect of anthocyanin concentrations on sperm survival rate in Zi geese. Data are expressed as the mean ± SD (*n* = 10). The dots with different lowercase letters represent statistically significant differences between the groups (*p* < 0.05); the dots with a common lowercase letter are not significantly different (*p* > 0.05).

In order to screen the optimal concentration of anthocyanin, anthocyanins (0, 10, 30, 50, 70, and 90 mg/L) were added to the semen diluent and then the semen of geese was diluted 100 times and stored at 37 C° for 6 h to detect sperm survival rate. As shown in Figure 2, there was a dose-dependent effect between the sperm survival rate and anthocyanin concentration when 10–30 mg/L of anthocyanins was added to the dilution solution. The highest sperm survival rate was observed at 30 mg/L, but there was no significant difference compared to the control group or 10 mg/L anthocyanin group (*p* > 0.05). The sperm survival rate of geese significantly decreased (*p* < 0.05) when the concentration of anthocyanins was increased to 50–90 mg/L, indicating an inhibitory effect on sperm survival rate. Therefore, 30 mg/L of anthocyanins was selected as the treatment concentration for subsequent experiments.

### 3.4. Effects of Anthocyanins on Semen Quality of Zi Geese

According to Table 3, the anthocyanin group showed a significant increase in sperm survival rate, sperm motility, plasma membrane integrity, and acrosome integrity (*p* < 0.05), while the sperm mortality rate decreased significantly (*p* < 0.05) compared with the control group. There were no significant differences in pH value, sperm density, or sperm deformity rate (*p* > 0.05).

### 3.5. Effects of Anthocyanins on Sperm Dynamics Parameters

According to Table 4, the VCL, VSL, and VAP of sperm in the anthocyanin group significantly increased (*p* < 0.05), while there was no significant effect on LIN (*p* > 0.05) compared with the control group.

### 3.6. The Effect of Anthocyanins on the Antioxidant Capacity of Goose Semen

According to the data presented in Table 5, notable differences in oxidative stress and antioxidant defense system indices were observed between the anthocyanin group and the control group. Compared with the control group, the anthocyanin group exhibited a significant increase in the contents of ROS and MDA (*p* < 0.05). Concomitantly, the activities of SOD and GPx were significantly reduced in the anthocyanin group relative to the control group (*p* < 0.05).

### 3.7. The Effect of Anthocyanins on Mitochondrial Activity of Goose Sperm

**Figure 3:**
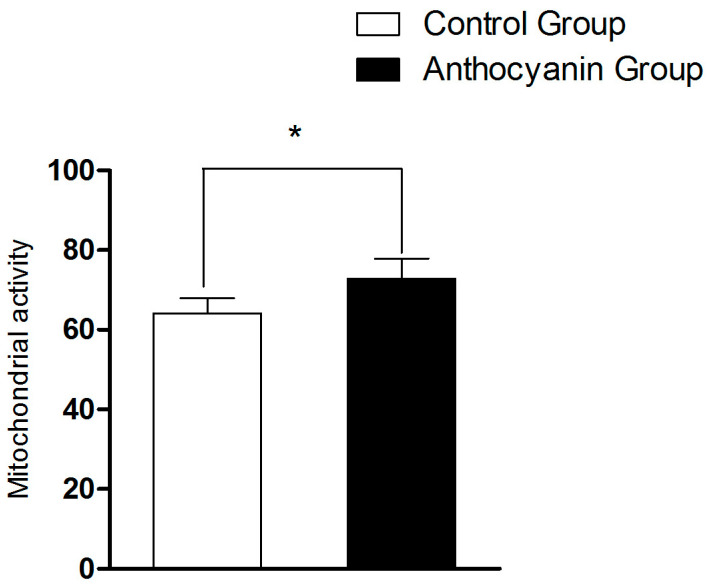
Effect of anthocyanin on mitochondrial activity of Zi goose sperm. Bars represent mean ± SD (*n* = 10). “*” represents a statistically significant difference between groups (*p* < 0.05).

As shown in Figure 3, the mitochondrial activity of the anthocyanin group was significantly increased (*p* < 0.05) compared with the control group.

### 3.8. Effects of Anthocyanins on Sperm Apoptosis in Zi Geese

**Figure 4:**
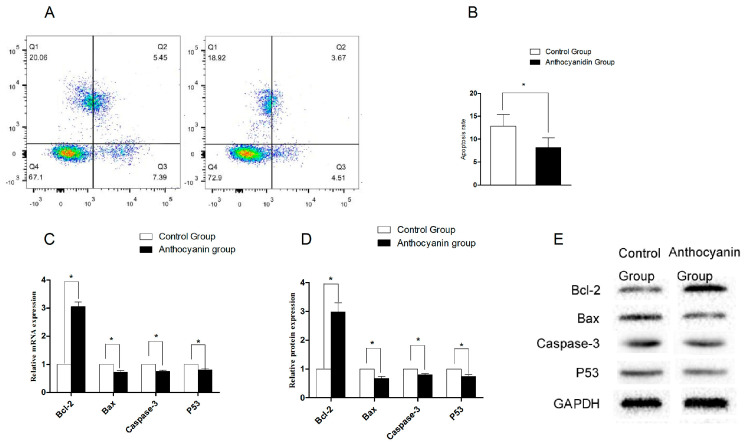
The effect of anthocyanin on sperm apoptosis in Zi geese. (**A**) Flow cytometry detection of sperm apoptosis. (**B**) The proportion of total apoptotic sperms. (**C**) Relative mRNA expression of *Bcl-2*, *Bax*, *Caspase-3*, and *P53* in the control group and anthocyanin group. (**D**,**E**) Relative protein expression of *Bcl-2*, *Bax*, *Caspase-3*, and *P53* in the control group and anthocyanin group. Bars represent mean ± SD (*n* = 10). “*” represents statistically significant differences between groups (*p* < 0.05).

As shown in Figure 4A,B, anthocyanins significantly decreased the apoptosis rate of sperm (*p* < 0.05). Moreover, anthocyanins significantly increased the mRNA and protein expression of *Bcl-2* (*p* < 0.05), and significantly decreased the mRNA and protein expression of *Bax*, *Caspase-3*, and *P53* (*p* < 0.05) compared with the control group (Figure 4C–E).

### 3.9. The Effect of Anthocyanins on DNA Fragmentation Rate of Goose Sperm

**Figure 5:**
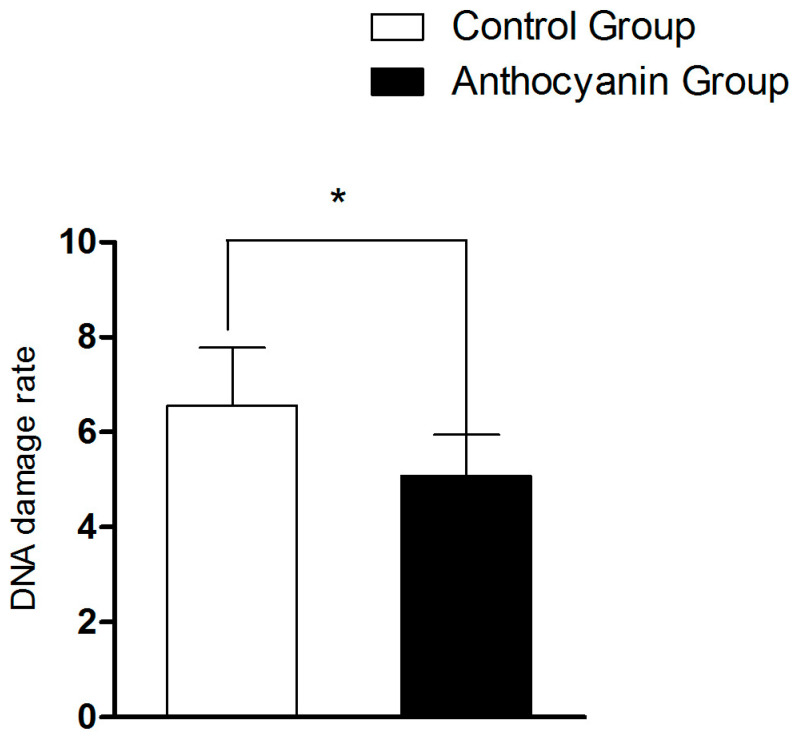
The effect of anthocyanin on DNA damage in Zi goose sperm. Bars represent mean ± SD (*n* = 10). “*” represents a statistically significant difference between groups (*p* < 0.05).

As shown in Figure 5, the DNA damage rate of the anthocyanin group was significantly decreased (*p* < 0.05) compared with the control group.

## 4. Discussion

This study systematically explored the effects of anthocyanins added to diluent on semen quality, semen antioxidant capacity, and sperm apoptosis. For the first time, the protective effect and mechanism of anthocyanins on the quality of Zi goose semen were revealed, providing multidimensional experimental basis for the development of natural protective agents for goose semen.

The core cause of the decline in semen quality during in vitro storage for Zi geese is the damage to the sperm membrane structure and the dysfunction of organelles [17]. Anthocyanins alleviate this process through a dual pathway of “physical protection + chemical regulation” [18]. The multiple phenolic hydroxyl groups contained in the molecular structure of anthocyanins can bind with unsaturated fatty acids in the phospholipid bilayer of sperm membranes, forming an “antioxidant barrier” that reduces ROS attacks on membrane lipids and lowers the degree of lipid peroxidation [4,19]. The MDA content in the 30 mg/L anthocyanin group was significantly decreased by 32.20% compared to the control group, directly confirming the protective effect of anthocyanins on membrane lipids. Meanwhile, anthocyanins can maintain membrane fluidity by regulating the ratio of cholesterol to phospholipids on the membrane surface [20]. This result was confirmed in the sperm motility parameters detected by CASA. The VCL, VSL, and VAP of sperm in the anthocyanin group were significantly increased compared to the control group, indicating that the improvement of sperm membrane fluidity directly enhanced sperm motility. Mitochondria are the core of sperm energy metabolism, and their structural integrity directly determines sperm vitality [21]. Mitochondria in the middle of the sperm tail produce ATP through oxidative phosphorylation, providing energy for sperm flagellar oscillation [22]. In this study, the mitochondrial activity of sperm in the anthocyanin group was significantly increased. The mechanism of this phenomenon was that anthocyanins can penetrate the mitochondrial membrane, clear ROS produced in the mitochondrial matrix, and inhibit the opening of mitochondrial permeability transition pores (mPTPs) [23]. The opening of mPTPs can lead to mitochondrial matrix efflux and a decrease in transmembrane potential (ΔΨm), which in turn can cause mitochondrial dysfunction [24]. In addition, anthocyanins can activate the SOD and GPx antioxidant systems in mitochondria, enhance their own antioxidant capacity, maintain oxidative phosphorylation efficiency, ensure sustained ATP supply, and safeguard sperm vitality from the energy source [25]. For the acrosome of sperm, the stability of its membrane structure determines the normal initiation of the acrosome reaction during fertilization [26]. In this study, the acrosome integrity rate of the anthocyanin group increased by 17.94% compared to the control group, which was closely related to the inhibition of premature activation of acrosome enzymes by anthocyanins. Acrosin is a serine protease, and excessive ROS can cause premature rupture of the acrosome membrane, leading to premature release of acrosin and loss of fertilization ability [27]. Anthocyanins improved semen quality by clearing ROS from semen, maintaining the closure of the acrosome membrane [8]. The phenomenon of decreased semen quality in the high-concentration anthocyanin group (50–90 mg/L) may be due to excessive phenolic hydroxyl groups disrupting the charge balance of the sperm membrane, leading to membrane protein denaturation and exacerbating membrane damage. This also suggests that the application of anthocyanins required strict control of concentration thresholds.

During the process of in vitro preservation of semen, oxidative stress is a key physiological process that leads to a decline in semen quality [28]. The mitochondrial respiratory chain of sperm in semen produces ROS. When the amount of ROS generated exceeds the clearance capacity of the antioxidant system of semen, it can trigger oxidative stress, attacking the sperm membrane, DNA, and enzyme active sites [29]. This study confirmed that anthocyanins rebuilt the oxidative antioxidant balance of semen through the synergistic effect of “directly clearing ROS + activating the antioxidant enzyme system”. Firstly, in the glycoside structure of anthocyanins, the ortho-dihydroxy group on the B ring has strong electron transfer ability and can act as a hydrogen donor to directly react with ROS, reducing them to harmless water molecules or oxygen, while being oxidized to a stable quinone structure [30]. In this study, the MDA content in the anthocyanin group was significantly lower than that in the control group, indicating that anthocyanins directly cleared ROS, reduced the accumulation of membrane lipid peroxidation products, and blocked the cascade reaction of oxidative stress from the source. Secondly, anthocyanins can enhance the antioxidant capacity of semen. SOD and GPx are the core antioxidant enzymes in semen. SOD is responsible for dismutation of O_2_^−^ into H_2_O_2_, while GPx further reduces H_2_O_2_ to H_2_O. In this study, the SOD and GPx activities of the anthocyanin group increased by 32.71% and 28.23% compared to the control group, respectively. Finally, oxidative stress not only directly damages the structure of sperm, but also leads to a decrease in enzyme activity and an imbalance of signaling molecules in semen. ROS can oxidize the ATPase in sperm, reducing the efficiency of ATP synthesis. Meanwhile, ROS can also disrupt the structure of calmodulin, affecting the calcium ion signaling pathway and leading to a decrease in sperm motility [31]. In this study, the improvement of sperm motility parameters in the anthocyanin group was related not only to membrane structure protection but also to the restoration of enzyme activity and stabilization of signaling pathways by anthocyanins. Anthocyanins can restore the active center structure of the enzyme through the thiol group (-SH) in the reductase molecule, protecting the selenocysteine residue in GPx from oxidation and ensuring its normal catalytic function [32].

Sperm apoptosis is another important reason for the decline in semen quality during in vitro preservation, and the mitochondrial apoptosis pathway is the core pathway that regulates sperm apoptosis, directly mediating the occurrence and execution of sperm apoptosis by releasing apoptotic factors and initiating caspases cascade reactions [33,34]. This study found that anthocyanins can significantly reduce the apoptosis rate of goose sperm and regulate the expression of apoptosis related genes through flow cytometry and qRT PCR detection, respectively. Anthocyanins block the initiation of apoptotic signals by maintaining the structural and functional integrity of mitochondria [35]. As previously mentioned, oxidative stress is the core trigger for inducing mitochondrial apoptosis. Excessive ROS can lead to a decrease in mitochondrial membrane potential and mPTP opening, releasing cytochrome C (CytC) from the mitochondrial matrix into the cytoplasm and initiating an apoptotic cascade reaction [36]. In this study, the mitochondrial activity of the anthocyanin group was higher than that of the control group, indicating that anthocyanins blocked the initiation of the mitochondrial apoptosis pathway from the source by inhibiting oxidative stress. In addition, anthocyanins can also regulate the balance of *Bcl-2* family proteins in mitochondria, and the ratio of *Bcl-2* to *Bax* is a key factor determining mitochondrial membrane permeability [37]. In this study, the *Bcl-2*/*Bax* ratio in the anthocyanin group was increased by 4.38 times compared to the control group. High expression of *Bcl-2* means it can easily bind with *Bax* to form heterodimers, inhibiting *Bax* from forming pores on the mitochondrial membrane, thereby preventing the release of CytC and further consolidating the anti-apoptotic ability of mitochondria [38]. Anthocyanins inhibit the activation of caspase family proteins and block the amplification of apoptotic signals [11]. When CytC is released into the cytoplasm, it binds to Apaf-1 to form an apoptosome, which activates *Caspase-9*. *Caspase-9* then activates *Caspase-3*, and *Caspase-3* can degrade structural and functional proteins within the cell, ultimately leading to cell apoptosis [39]. In this study, the expression of *Caspase-3* mRNA and protein in the anthocyanin group were significantly lower than those in the control group, indicating that anthocyanins can inhibit the activation of *Caspase-3*. In addition, anthocyanins can also inhibit the degradation of sperm tail actin by *Caspase-3*, maintain the structural integrity of sperm flagella, and ensure that sperm still have certain motility [13], which was also one of the important reasons for the improvement of sperm vitality in the anthocyanin group. Gao et al. found that anthocyanins can bind to DNA to form complexes, protecting DNA from degradation mediated by ROS and caspase and reducing DNA fragmentation [40]. In this study, the sperm DNA integrity rate of the anthocyanin group was significantly increased compared to the control group. Anthocyanins protect sperm DNA and structural proteins, reduce apoptosis related cell damage, and reduce the impact of apoptosis on sperm, confirming the protective effect of anthocyanins on goose sperm DNA.

## 5. Conclusions

Under the conditions of this experiment, the addition of 30 mg/L of anthocyanins to the semen diluent is the dose threshold for improving the semen quality of Zi geese. Specifically, 30 mg/L of anthocyanins added to the diluent can significantly increase sperm survival rate, sperm motility, plasma membrane integrity rate, and acrosome integrity rate, while concurrently reducing sperm mortality rate and DNA damage. Anthocyanins can directly scavenge ROS in Zi goose semen, reduce the accumulation of MDA, enhance the activities of SOD and GPx, and thereby strengthen the antioxidant defense ability of semen. Furthermore, anthocyanins inhibited sperm apoptosis by regulating the mitochondrial apoptosis pathway. Collectively, anthocyanins can act as a natural protectant for Zi goose semen and have broad application prospects in the in vitro preservation and artificial insemination practices of Zi goose semen.

## Figures and Tables

**Table 1:** Composition of semen diluent.

Ingredient	Content
Fructose (g)	1.000
Magnesium chloride (g)	0.068
Sodium acetate (g)	0.857
Potassium citrate (g)	0.128
Sodium glutamate (g)	1.920
Penicillin (IU)	100,000
Streptomycin (IU)	100,000
Distilled water (mL)	100

**Table 2:** Primers used in this study.

Gene	Accession Number	Primer Sequence	Product Size (bp)
*Bcl-2*	KM040761	F: 5′-GAGTACCTGAACCGGCATCT-3′ R: 5′-CAGCCTCCGTTATCCTGGTA-3′	151
*Bax*	XM_066985593	F: 5′-GGAATTCGCCGAGATGTCCG-3′ R:5′-CGGGATCCGGTCTGATCGTCG-3′	129
*Caspase-3*	XM_066996225	F: 5′-ATGGCCCTGGACATTGAC-3′ R: 5′-TTGCTGTCGCTGCTTGT-3′	135
*P53*	XM_013175338	F: 5′-GAGTCTGCCCGGACGAT-3′ R: 5′-TCCTCGGTGCTCGATGT-3′	145

**Table 3:** Effects of anthocyanin on semen quality of Zi geese.

	Control Group	Anthocyanin Group	
pH value	7.32 ± 0.54	7.20 ± 0.63	
Sperm concentration (pcs/mL)	3.85 ± 0.29	3.77 ± 0.16	
Sperm motility	0.70 ± 0.05 ^b^	0.76 ± 0.03 ^a^	
Sperm survival rate (%)	76.98 ± 5.67 ^b^	81.08 ± 3.85 ^a^	
Sperm abnormality rate (%)	8.71 ± 0.64	8.62 ± 0.71	
Sperm mortality rate (%)	23.02 ± 1.68 ^a^	18.92 ± 2.34 ^b^	
Membrane integrity rate (%)	67.56 ± 2.60 ^b^	72.63 ± 3.09 ^a^	
Acrosome integrity rate (%)	78.80 ± 4.52 ^b^	92.94 ± 4.67 ^a^	

Note: Data are expressed as the mean ± SD (*n* = 10). In the same row, different lowercase superscripts indicate significant difference (*p* < 0.05), and the absence of lowercase superscripts indicates insignificant difference (*p* > 0.05).

**Table 4:** Effects of anthocyanin on sperm dynamics parameters.

	Control Group	Anthocyanin Group
VCL (μm/s)	103.87 ± 6.22 ^b^	118.84 ± 7.32 ^a^
VSL (μm/s)	68.46 ± 8.13 ^b^	72.83 ± 7.20 ^a^
VAP (μm/s)	80.63 ± 6.38 ^b^	89.91 ± 5.48 ^a^
LIN (%)	63.44 ± 5.47	62.98 ± 8.01

Note: Data are expressed as the mean ± SD (*n* = 10). In the same row, different lowercase superscripts indicate significant difference (*p* < 0.05), and the absence of lowercase superscripts indicate insignificant difference (*p* > 0.05).

**Table 5:** Effects of anthocyanin on oxidative stress indicators in Zi goose semen.

	Control Group	Anthocyanin Group
ROS (OD Value)	0.63 ± 0.04 ^a^	0.36 ± 0.03 ^b^
MDA (nmol/L)	7.64 ± 0.81 ^a^	5.18 ± 0.72 ^b^
SOD (U/mL)	166.07 ± 12.38 ^b^	220.39 ± 11.84 ^a^
GPx (U/L)	118.55 ± 8.61 ^b^	152.02 ± 13.46 ^a^

Note: Data are expressed as the mean ± SD (*n* = 10). In the same row, different lowercase superscripts indicate significant difference (*p* < 0.05).

## Data Availability

Data are available upon request.

## Supplementary Materials

The following supporting information can be downloaded at: https://www.mdpi.com/article/10.3390/ani15223281/s1, Table S1. Antibody information and incubation conditions. Table S2. Results of goose semen collection.
