# Supplementary material for: The Effects of Anthocyanins Added to Semen Diluent on Semen Quality, Semen Antioxidant Capacity, and Sperm Apoptosis in Zi Geese
Source: Animals (Basel). 2025 Nov 13;15(22):3281. doi: 10.3390/ani15223281 (PMC12649422; doi:10.3390/ani15223281)
Supplement: Supplementary file 1 [file animals-15-03281-s001.zip › animals-3986616-supplementary.pdf]

Table S1 Antibody information and incubation conditions

Antibodies	Cat. No.	Diluted ratio	Loading quantity of protein sample	Incubation conditions	Exposure time
Bcl-2	LM-1353R	1: 1,000	10 μg	4 °C overnight	10 s-30 s
Bax	TFC BS-0127R	1: 1,000	10 μg	4 °C overnight	10 s-30 s
Caspase-3	Ab 13847	1: 1,000	10 μg	4 °C overnight	10 s-30 s
P53	NB 100-92306	1: 1,000	10 μg	4 °C overnight	10 s-30 s
HRP labeled secondary antibody	Ab 125856	1: 5,000	-	Room temperature for 1 h	-

Table S2 Results of goose semen collection

Total number of geese	The number of geese that have collected semen	The proportion of goose with semen collected to the total number (%)	Semen volume (mL)
60	55	91.67	0.35 ± 0.01
